# Teacher support and L2 writing engagement in GenAI-integrated classrooms: a serial mediation model of growth mindset and needs satisfaction

**DOI:** 10.3389/fpsyg.2026.1762106

**Published:** 2026-02-18

**Authors:** Yanxiao Chen, Jaewoo Shim

**Affiliations:** Department of English Education, College of Education, Jeonbuk National University, Jeonju, Republic of Korea

**Keywords:** GenAI-integrated classrooms, growth mindset, L2 writing engagement, needs satisfaction, teacher support

## Abstract

**Introduction:**

Teacher support is a well-established predictor of L2 writing engagement. However, the mechanisms through which it operates remain insufficiently understood, particularly in classrooms where Generative AI (GenAI) tools are integrated into instruction. Drawing on Self-determination theory (SDT) and Control–value theory (CVT), this study conceptualizes teacher support as the environmental input, growth mindset as a control appraisal, and needs satisfaction as the motivational state closely linked to engagement.

**Methods:**

Survey data were collected from 366 Chinese university EFL learners enrolled in GenAI-integrated L2 writing classrooms and analyzed using structural equation modeling.

**Results:**

Results showed that all three dimensions of teacher support and growth mindset were positively associated with needs satisfaction, and that both growth mindset and needs satisfaction positively predicted L2 writing engagement. Competence support was linked to engagement through separate pathways involving needs satisfaction and growth mindset, as well as through a sequential growth-mindset → needs-satisfaction mechanism. Emotional and autonomy support showed significant direct associations with engagement and indirect effects via needs satisfaction.

**Discussion:**

These findings provide initial evidence for an SDT–CVT–aligned process, in which higher levels of teacher support are associated with higher engagement through appraisal-driven motivational states within GenAI-integrated instructional contexts. This offers practical guidance for sustaining learner engagement in GenAI-integrated L2 writing.

## 1 Introduction

L2 writing engagement is widely recognized as a central factor associated with writing development, encompassing learners’ behavioral effort, cognitive investment, emotional involvement, and agentic participation (Fredricks et al., 2004; Li and Yuan, 2024; Rahimi, 2024; Zhou and Hiver, 2022). Engaged writers tend to revise more extensively, apply more effective strategies, persist longer, and ultimately achieve higher-quality writing outcomes (Hiver et al., 2020; Zhou and Hiver, 2022). However, disengagement—characterized by procrastination, superficial revision, and low cognitive involvement—remains pervasive in many university classrooms (Zhou and Hiver, 2022). Consequently, identifying contextual and psychological factors that sustain L2 writing engagement has become a key concern across applied linguistics and educational psychology.

The rapid diffusion of Generative Artificial Intelligence (GenAI) tools such as ChatGPT, DeepSeek, and Gemini has increased the availability of GenAI-mediated feedback and assistance in L2 writing instruction. GenAI applications can offer rapid feedback, language explanations, and alternative phrasings that facilitate iterative drafting and strategy refinement (Kasneci et al., 2023; Mekheimer, 2025; Yan, 2024). At the same time, overreliance on GenAI-generated text, limited prompt literacy, and uncertainty regarding the accuracy and appropriateness of GenAI suggestions can undermine self-regulation or obscure instructional goals (Kim et al., 2025; Zhai et al., 2024). In GenAI-integrated L2 writing classrooms, teacher support therefore continues to play an important role in shaping learners’ motivation and engagement, as teachers help students navigate the opportunities and risks of human–GenAI collaborative writing (Yan and Zhang, 2024; Zare et al., 2025). Despite growing research on GenAI-supported L2 writing, existing studies have predominantly focused on the affordances and classroom applications of GenAI tools, with limited exploration of the broader instructional ecology in which they are situated (Asad et al., 2024; Kasneci et al., 2023; Wang and Xue, 2024). At the same time, research on teacher support, learner motivation, and engagement in writing or language learning contexts has largely developed independently of GenAI integration (e.g., Sadoughi and Hejazi, 2023; Yin et al., 2025). As a result, how teacher support operates within GenAI-integrated writing classrooms to be associated with learners’ motivational processes and engagement remains insufficiently understood.

Self-determination theory (SDT) provides a foundational account of how social-contextual factors energize engagement by satisfying autonomy, competence, and relatedness needs (Deci and Ryan, 2000; Ryan and Deci, 2023). Need-supportive teacher practices are therefore expected to facilitate sustained engagement (Reeve, 2012). Yet SDT provides a limited explanation of the cognitive appraisals that precede motivational states. Control–value theory (CVT) addresses this gap by proposing that environmental inputs are proposed to relate to learners’ appraisals—such as perceived controllability and beliefs about ability—which, in turn, are related to motivation and engagement (Pekrun, 2006; Pekrun and Linnenbrink-Garcia, 2012; Pekrun and Perry, 2014). Integrating SDT and CVT thus enables a richer account of how teacher support may be linked to engagement: environment → appraisal → motivational state → engagement. A key appraisal in L2 writing is growth mindset—the belief that writing ability is malleable and can be improved through effort, strategy use, and constructive feedback (Dweck, 2006). Growth-oriented learners are more likely to persist through revision, respond adaptively to errors, and productively engage with teacher and GenAI-generated feedback (Fathi et al., 2024; Lou and Noels, 2019).

Taken together, SDT and CVT suggest a coherent motivational sequence through which teacher support may be positively associated with L2 writing engagement: teacher support → growth mindset → needs satisfaction → engagement. Empirical research, however, has not sufficiently examined these mechanisms jointly, particularly in classrooms where learners interact with both human and GenAI resources. To address this gap, the present study proposes a serial mediation model in which teacher support is associated with L2 writing engagement by influencing growth mindset and needs satisfaction. GenAI is conceptualized as a contextual instructional condition rather than a focal analytic variable; therefore, the present study addresses motivational processes operating in GenAI-integrated classrooms rather than effects that can be causally attributed to GenAI use per se.

## 2 Literature review

### 2.1 Theoretical framework

The present framework specifies the analytic roles and sequential ordering of key constructs in a serial mediation model of L2 writing engagement. Within this model, perceived teacher support functions as the initiating contextual input, capturing learners’ interpretations of instructional conditions that structure and support L2 writing. Growth mindset is positioned as an intermediate cognitive mechanism, reflecting learners’ appraisals of writing ability as improvable and shaping how instructional support is interpreted and acted upon during writing and revision. Basic psychological needs satisfaction is specified as a proximal motivational state, representing learners’ experienced competence, autonomy, and relatedness while engaging in writing tasks. Accordingly, the framework delineates a directional motivational sequence in which teacher support is hypothesized to predict growth mindset, which in turn facilitates needs satisfaction and ultimately relates to sustained L2 writing engagement. GenAI is treated as a contextual condition of instruction, within which these motivational processes operate, rather than as an analytic predictor.

### 2.2 Perceived teacher support and L2 writing engagement

Based on previous research on teacher support (Federici and Skaalvik, 2014) and SDT (Deci and Ryan, 2000), the present study conceptualizes teacher support as competence, emotional, and autonomy support. These three dimensions align with learners’ basic psychological needs for competence, relatedness, and autonomy, and collectively shape L2 learners’ motivation and engagement (Lietaert et al., 2015). In L2 writing, where tasks impose heavy cognitive, affective, and strategic demands, these forms of support play a particularly critical role (Zhou and Hiver, 2022).

Competence support refers to teacher practices that strengthen learners’ perceptions of capability and progress by providing clear explanations, structured guidance, and process-oriented feedback that foster independent and critical thinking (Li and Chiu, 2025; Yin et al., 2025). In L2 writing, such support is evident in teachers’ efforts to clarify task expectations, model genre and rhetorical conventions, and scaffold learners’ planning and revision processes—practices that help students manage the substantial cognitive demands of writing (Ahn, 2012; Han and Hyland, 2015; Hyland, 2007). These practices remain essential even when GenAI tools are available, as students must critically evaluate GenAI-generated suggestions rather than adopt them unreflectively.

Emotional support encompasses teachers’ expressions of care, empathy, and encouragement that foster a trusting and psychologically safe classroom climate (Ahmadi et al., 2023; Zhou et al., 2025). In writing contexts, emotional support enables students to express ideas freely, ask questions without fear of negative evaluation, and maintain constructive teacher–student relationships, all of which help learners feel cared for and noticed (Zhang and Jiang, 2025). Emotional support may be particularly salient in GenAI-mediated writing, as students can experience uncertainty about the appropriateness or accuracy of GenAI tools’ feedback.

Autonomy support involves teacher behaviors that nurture learners’ sense of volition, choice, and agency (Deci and Ryan, 2000). In L2 writing, this includes encouraging students to make independent decisions regarding writing topics, planning strategies, and revision choices, thereby enabling them to take ownership of their writing development (Vo, 2023; Yeung, 2019). Within GenAI-integrated classrooms, autonomy support also includes guiding learners to use GenAI tools responsibly, critically evaluating GenAI tools’ suggestions, and integrating them into self-directed revision practices.

Substantial evidence from traditional classrooms confirms that teacher support is a robust predictor of L2 learning engagement (Dincer et al., 2019; Reeve, 2013; Sadoughi and Hejazi, 2023). Competence and emotional support enhance engagement by strengthening self-efficacy and positive achievement goals (Liu et al., 2023), while autonomy support directly promotes enjoyment, task value, and sustained effort (Vo, 2023). Despite this evidence, few studies have disentangled the unique contributions of each support dimension within the specific domain of L2 writing. Emerging research suggests that GenAI can offer personalized guidance, mitigate uncertainty, and facilitate engagement in drafting and revision (Li and Chiu, 2025; Mekheimer, 2025; Yan, 2024). For instance, Zare et al. (2025) found that ChatGPT integration enhanced students’ sense of partnership and reduced anxiety, thereby increasing writing engagement. However, these studies primarily illuminate the technology’s affordances. This leaves a critical question unanswered: In a classroom where students have access to powerful GenAI tools, how does teacher support continue to shape L2 writing engagement?

Because L2 writing involves complex cognitive regulation and recursive decision making that are reshaped when learners collaborate with GenAI tools, it is timely and theoretically important to clarify how each dimension of teacher support uniquely contributes to learners’ motivation in this new environment.

### 2.3 Growth mindset and needs satisfaction as potential mediators

In Dweck’s (2006) framework, a growth mindset reflects the view that one’s abilities in a given domain can develop over time through sustained effort, practical strategies, and feedback. As a cognitive appraisal that shapes how learners interpret difficulty, error, and feedback, the growth mindset has received increasing attention in contemporary educational psychology and L2 research (Zhang, 2024). Growth-oriented beliefs typically develop through experiences that foreground progress, opportunities to overcome meaningful challenges, and social messages, particularly from teachers, that emphasize improvement and effort (Dweck and Yeager, 2019; Vestad and Bru, 2024). In L2 learning, a strong growth mindset is associated with greater motivation, stronger self-efficacy, and deeper behavioral, cognitive, and emotional engagement (Lou and Noels, 2019). Recent evidence extends these findings to L2 writing, demonstrating positive links between growth mindset, writing enjoyment, ideal L2 self, and writing engagement (Fathi et al., 2024; Yao et al., 2024).

Beyond its well-documented motivational benefits, emerging studies suggest that a growth mindset may also facilitate learners’ basic psychological needs. For example, Gu and Wang (2024) identified positive associations between growth mindset and basic needs satisfaction among Chinese adolescents and university students. Similarly, Pan and Li (2025) reported that students with stronger growth mindsets demonstrated greater well-being and psychological need fulfillment in GenAI-integrated learning contexts. These findings suggest that growth-oriented beliefs may support needs satisfaction by encouraging adaptive responses, effortful engagement, and constructive interpretations of setbacks.

Teacher support plays a central role in cultivating such beliefs. Process-focused and improvement-oriented feedback, such as highlighting progress, modeling strategies, and framing difficulty as natural and surmountable, helps learners internalize the idea that writing ability can be strengthened over time (Dweck, 2006; Reeve, 2012). When students adopt these beliefs, they tend to revise more intentionally, seek clarification proactively, and interpret errors as informative rather than discouraging (Park et al., 2020). Empirical studies show that growth-mindset interventions increase learners’ persistence, cognitive involvement, and L2 writing engagement (Fathi et al., 2024). These findings position growth mindset as a theoretically meaningful mediator through which teacher support fosters deeper engagement in L2 writing.

From an SDT standpoint, learners experience more self-determined motivation and sustained engagement when their needs for autonomy, competence, and relatedness are adequately met (Deci and Ryan, 2000; Ryan and Deci, 2023). Teacher support predicts needs satisfaction by offering meaningful choices, constructive guidance, and socioemotional encouragement (He et al., 2025). In L2 writing, autonomy and competence satisfaction foster willingness to revise, experimentation with linguistic forms, and deeper involvement in drafting and feedback cycles. Related evidence also shows that competence-related emotions support engagement in writing tasks (Sadoughi and Hejazi, 2023).

Together, the literature suggests that growth mindset and needs satisfaction may function as sequential mediators linking teacher support to L2 writing engagement, a pathway that this study empirically examines.

### 2.4 Present study

**Figure 1:**
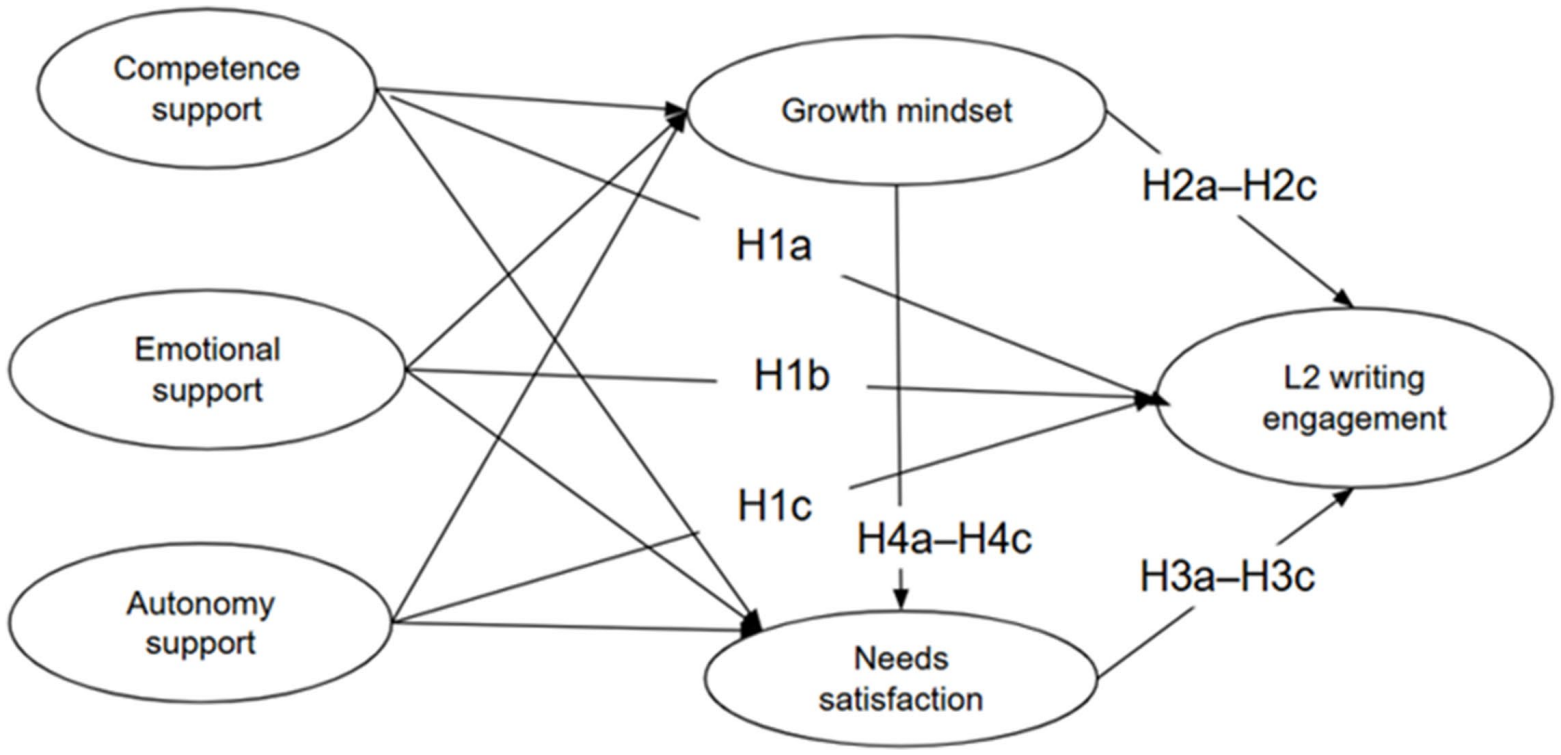
The hypothesized research model.

Drawing on SDT and CVT, the present study examines how teacher support enhances L2 writing engagement in GenAI-integrated classrooms. Specifically, it investigates how competence, emotional, and autonomy support influence engagement directly and indirectly through growth mindset and needs satisfaction. Accordingly, the study formulates the following hypotheses (see Figure 1):

*H1*: The three dimensions of teacher support (competence H1a, emotional H1b, and autonomy H1c) are expected to predict L2 writing engagement positively.

*H2*: Growth mindset is expected to mediate the relationships linking competence (H2a), emotional (H2b), and autonomy (H2c) support to L2 writing engagement.

*H3*: Needs satisfaction is expected to mediate the relationships linking competence (H3a), emotional (H3b), and autonomy (H3c) support to L2 writing engagement.

*H4*: The effects of competence (H4a), emotional (H4b), and autonomy (H4c) support on L2 writing engagement are expected to operate sequentially through growth mindset and, subsequently, needs satisfaction.

## 3 Methods

### 3.1 Participants

A total of 400 Chinese university students enrolled in English writing courses were recruited through Credamo, a widely used online data-collection platform. In this study, GenAI-integrated instruction was operationally defined as English writing courses in which students reported that GenAI tools had been explicitly introduced by the instructor and were required or strongly encouraged for at least one major writing task within the preceding 3 months. This definition was based on students’ self-reported perceptions of instructional practices, which aligns with the study’s focus on perceived teacher support and learners’ motivational experiences rather than on objectively verified instructional policies or usage logs. Participation was voluntary and anonymous. Before completing the questionnaire, students were informed of the study’s purpose, procedures, confidentiality safeguards, and their right to withdraw at any point. Electronic informed consent was obtained, and participants received a small incentive upon completion of the study.

After applying data-quality screening criteria, 366 valid responses were retained (effective response rate = 91.5%). Cases were excluded if participants (a) failed the attention-check item, (b) provided invariant responses across all items, or (c) completed the survey in less than one-third of the median completion time, consistent with recommended thresholds for identifying inattentive or automated responding (DeSimone and Harms, 2018; Meade and Craig, 2012). The final sample comprised 196 females (53.56%) and 170 males (46.44%) from diverse disciplinary backgrounds, including the liberal arts, sciences, engineering, and other related fields. Participants ranged in age from 17 to 22 years (*M* = 19.27), reflecting the demographic profile commonly reported for Chinese university learners of L2 writing. Because participants were recruited from multiple institutions and instructional contexts, teacher support was conceptualized and analyzed as an individual-level perceived construct rather than a shared classroom-level variable.

### 3.2 Instruments

A structured questionnaire was used to collect the data. It comprised two main sections: (a) background information (e.g., gender, age, and academic discipline) and (b) focal measures assessing three forms of teacher support, learners’ growth mindset, need satisfaction, and L2 writing engagement, along with two control variables—GenAI literacy and GenAI attitudes. All items were rated on a five-point Likert scale (1 = strongly disagree to 5 = strongly agree). The original instruments have shown good internal consistency in prior research and were adapted for use in the GenAI-supported L2 writing context. To ensure linguistic and contextual appropriateness for GenAI-supported L2 writing classrooms, the instruments were adapted for this study and translated into Chinese using a rigorous translation/back-translation process. Two bilingual specialists produced independent forward translations and reconciled any divergence through discussion. A third expert then back-translated the Chinese version into English to confirm semantic alignment with the original items. Prior to administering the formal survey, the draft questionnaire was tested on 30 EFL learners to assess item clarity, resulting in minor revisions. The subsequent subsections provide a detailed introduction to each measurement scale. Across all measures, GenAI integration was treated as a contextual instructional condition based on students’ perceptions of classroom practices, rather than as an analytic variable capturing specific tools, usage frequency, or instructional designs.

#### 3.2.1 Perceived teacher support

Teacher support was operationalized as students’ perceived instructional and interpersonal support, reflecting an individual-level psychological construct rather than a shared classroom-level characteristic. Teacher support consisted of three dimensions, competence support, emotional support, and autonomy support, adapted from Standage et al. (2005), Yin et al. (2025), and Li and Chiu (2025). Competence support was measured using three items with an original reliability of *α* = 0.84; a sample item is: “My English teacher made me feel that I was able to do the writing activities.” Emotional support was assessed with three items (*α* = 0.88), such as “My L2 writing teacher was friendly to me.” Autonomy support was measured using three items (*α* = 0.92), for example, “My teacher allows me to write at my own pace.”

#### 3.2.2 Growth mindset

Growth mindset was measured using six items adapted from Bai and Wang (2021) and Yao et al. (2024), which focus on beliefs about the malleability of L2 writing ability. A sample item is: “If I practice more, I can improve my English writing competence.” In previous research, the scale demonstrated good internal consistency (α = 0.89; Yao et al., 2024).

#### 3.2.3 Needs satisfaction

Learners’ basic psychological needs satisfaction, encompassing autonomy, competence, and relatedness, was assessed using items adapted from Chiu (2024) and Li and Chiu (2025), with acceptable original reliabilities (*α* > 0.71). Autonomy satisfaction was measured with three items, for example: “I feel a sense of choice and freedom in the L2 writing process.” Competence satisfaction was assessed with three items, such as “I feel competent to achieve my writing goals.” Relatedness satisfaction was measured with three items, for instance: “I feel close and connected to my writing activities, which are important to me.”

#### 3.2.4 L2 writing engagement

L2 writing engagement comprised four subdimensions—behavioral, emotional, cognitive, and agentic—adapted from frameworks proposed by Guo et al. (2025), Reeve (2013), Skinner et al. (2009), and Wang et al. (2016). Behavioral engagement was measured with three items with acceptable original reliability (*α* > 0.77), for example: “I participate in all the activities in the writing class.” Emotional engagement included three items with acceptable original reliability (α > 0.80), such as “I enjoy learning new things in English writing.” Cognitive engagement was assessed with three items (α > 0.85), for instance: “I go through my writing carefully to make sure it is correct.” Agentic engagement was measured with three items with acceptable reliability (α = 0.84), for example: “I express my preferences and opinions during the writing class.”

#### 3.2.5 GenAI literacy and GenAI attitudes (control variables)

The following two scales were used as control variables. GenAI literacy was assessed using a brief, domain-specific scale adapted from Xu et al. (2025), which captures learners’ perceived ability to understand, evaluate, and responsibly use GenAI tools in L2 writing. A sample item is: “I can judge whether GenAI-generated suggestions are appropriate for my writing goals.” GenAI attitudes were measured using the validated GenAI Attitude Scale (AIAS-4) (Grassini, 2023), which assesses learners’ general evaluative orientation toward artificial intelligence. A sample item is: “I think GenAI technology is positive for humanity.” In the present study, the GenAI literacy scale demonstrated acceptable internal consistency (α = 0.86), and the GenAI attitudes scale also showed good reliability (α = 0.79). Both GenAI literacy and GenAI attitudes were included as control variables in the hierarchical regression analyses to examine the robustness of the proposed motivational model.

### 3.3 Data collection and analysis

Data were collected through Credamo, a professional online data-collection platform widely used in academic research in China. Credamo employs verified participants and a built-in quality control system. Based on pre-specified screening criteria (i.e., university-level EFL learners), the platform distributed the questionnaire to the target participant group.

Data analysis was conducted using SPSS 27 and AMOS 27. To examine whether common method variance posed a threat to the results, Harman’s one-factor procedure was conducted (Podsakoff et al., 2003) by submitting all measurement items to an unrotated exploratory factor analysis. The first emerging factor explained 28.12% of the total variance—well below the commonly referenced 40% benchmark—suggesting that common method bias was unlikely to distort the findings. Reliability and validity were assessed in line with established guidelines (Byrne, 2013; Pallant, 2020). SPSS 27 was used to compute descriptive statistics (means, standard deviations, skewness, and kurtosis), and bivariate correlations to evaluate the distributional characteristics of the variables and their interrelationships, including teacher support, growth mindset, needs satisfaction, and L2 writing engagement. Structural paths were then estimated in AMOS 27 to evaluate the proposed mediation model, testing whether growth mindset and needs satisfaction mediated the effects of teacher support on L2 writing engagement. Indirect effects were examined through bias-corrected bootstrapping with 95% confidence intervals; mediation was considered present when the confidence interval did not include zero (Hayes, 2017). To examine the robustness of the proposed model, GenAI literacy and GenAI attitudes were included as control variables in supplementary hierarchical regression analyses. Because the present study examined motivational processes within GenAI-integrated instructional contexts, it did not aim to quantify GenAI usage intensity, compare specific GenAI tools, or evaluate the quality of teacher guidance around GenAI use.

## 4 Results

### 4.1 Descriptive statistics

Table 1 reports the descriptive statistics for all study variables. Learners perceived moderately high levels of teacher support, including competence support (*M* = 3.30), emotional support (*M* = 3.714), and autonomy support (*M* = 3.746). Growth mindset also showed a moderate mean level (*M* = 3.509), suggesting that students generally believed in the improvability of their L2 writing ability. Needs satisfaction (*M* = 3.745) and L2 writing engagement (*M* = 3.698) were similarly moderate to high, indicating that learners felt reasonably supported in their autonomy, competence, and relatedness needs and were generally willing to invest effort into writing tasks.

**Table 1:** Descriptive statistics.

Variables	*M*	SD	Skewness	Kurtosis	*α*
CTS	3.300	0.994	−0.516	−0.806	0.852
ETS	3.714	0.924	−0.581	−0.424	0.830
ATS	3.746	0.839	−0.698	−0.369	0.760
GM	3.509	1.003	−0.430	−0.902	0.903
NS	3.745	0.714	−0.489	−0.590	0.862
L2WE	3.698	0.739	−0.399	−0.632	0.909

CTS, competence teacher support; ETS, emotional teacher support; ATS, autonomy teacher support; GM, growth mindset; NS, needs satisfaction; L2WE, L2 writing engagement.

In the present sample, all scales demonstrated satisfactory internal consistency, with Cronbach’s *α* ranging from 0.760 to 0.909, exceeding the recommended threshold of 0.70. Skewness and kurtosis values fell within the recommended thresholds of ±2 for normality (Kline, 2023), supporting the use of maximum likelihood estimation.

Correlation analysis (Table 2) revealed that the three dimensions of teacher support were differentially associated with growth mindset. Competence support showed a weak but significant correlation with growth mindset (*r* = 0.226, *p* < 0.01), whereas emotional support (*r* = 0.060, *p* > 0.05) and autonomy support (*r* = 0.056, *p* > 0.05) were not significantly related. In contrast, all three types of teacher support were significantly associated with needs satisfaction (*r* = 0.428, 0.283, and 0.306; all *p* < 0.01) and L2 writing engagement (*r* = 0.303, 0.295, and 0.382; all *p* < 0.01). Growth mindset demonstrated moderate positive correlations with both needs satisfaction (*r* = 0.337, *p* < 0.01) and L2 writing engagement (*r* = 0.443, *p* < 0.01). Needs satisfaction showed the strongest association with L2 writing engagement (*r* = 0.519, *p* < 0.01). All correlations were below 0.52, well under commonly cited thresholds for multicollinearity (*r* > 0.70), indicating no multicollinearity concerns.

**Table 2:** Factor loadings and correlation matrix for all variables.

Variables	FL	CR	CTS	ETS	ATS	GM	NS	L2WE
CTS	0.819	0.860	**0.821**					
ETS	0.800	0.844	0.278**	**0.802**				
ATS	0.750	0.795	0.204**	0.194**	**0.751**			
GM	0.785	0.907	0.226**	0.060	0.056	**0.787**		
NS	0.690	0.741	0.428**	0.283**	0.306**	0.337**	**0.698**	
L2WE	0.710	0.802	0.303**	0.295**	0.382**	0.443**	0.519**	**0.710**

CR, composite reliability; FL, factor loading. Factor loadings represent mean standardized loadings for indicators of each latent construct. Factor loading ranges were as follows: CTS (0.765–0.885), ETS (0.732–0.861), ATS (0.726–0.798), GM (0.713–0.876), NS (0.691–0.702), and L2WE (0.701–0.711). Diagonal values (in bold) represent the square root of the average variance extracted (AVE). **Indicates *p* < 0.01. CTS, competence teacher support; ETS, emotional teacher support; ATS, autonomy teacher support; GM, growth mindset; NS, needs satisfaction; L2WE, L2 writing engagement.

### 4.2 Measurement model

A confirmatory factor analysis was conducted to evaluate the measurement properties of the six latent constructs: competence teacher support (CTS), emotional teacher support (ETS), autonomy teacher support (ATS), growth mindset (GM), needs satisfaction (NS), and L2 writing engagement (L2WE). Teacher support was operationalized as three distinct but correlated dimensions. Growth mindset and needs satisfaction were mediators. L2 writing engagement was modeled as a multidimensional construct (behavioral, cognitive, emotional, and agentic engagement). Following recommendations by Little et al. (2002, 2013), item parcels were created by averaging items within theoretically coherent subdimensions (e.g., behavioral vs. emotional engagement). This approach reduces idiosyncratic item variance, improves indicator reliability, and yields a more parsimonious model that does not obscure the underlying factor structure.

The measurement model demonstrated a good fit to the data, with *χ^2^*/df = 2.088, CFI = 0.945, TLI = 0.934, RMSEA = 0.055, and SRMR = 0.045, all of which fall within the recommended guidelines (e.g., Kline, 2023). All standardized factor loadings exceeded 0.60 (*p* < 0.001), supporting strong indicator reliability. Composite reliability (CR) ranged from 0.741 to 0.907, indicating acceptable to excellent internal consistency. Discriminant validity was supported, as the square root of each construct’s AVE was greater than its correlations with all other constructs (Henseler et al., 2015). These results demonstrate that the latent constructs were measured reliably and distinctly, providing a robust foundation for testing the structural model.

### 4.3 Model fit and path analysis

**Figure 2:**
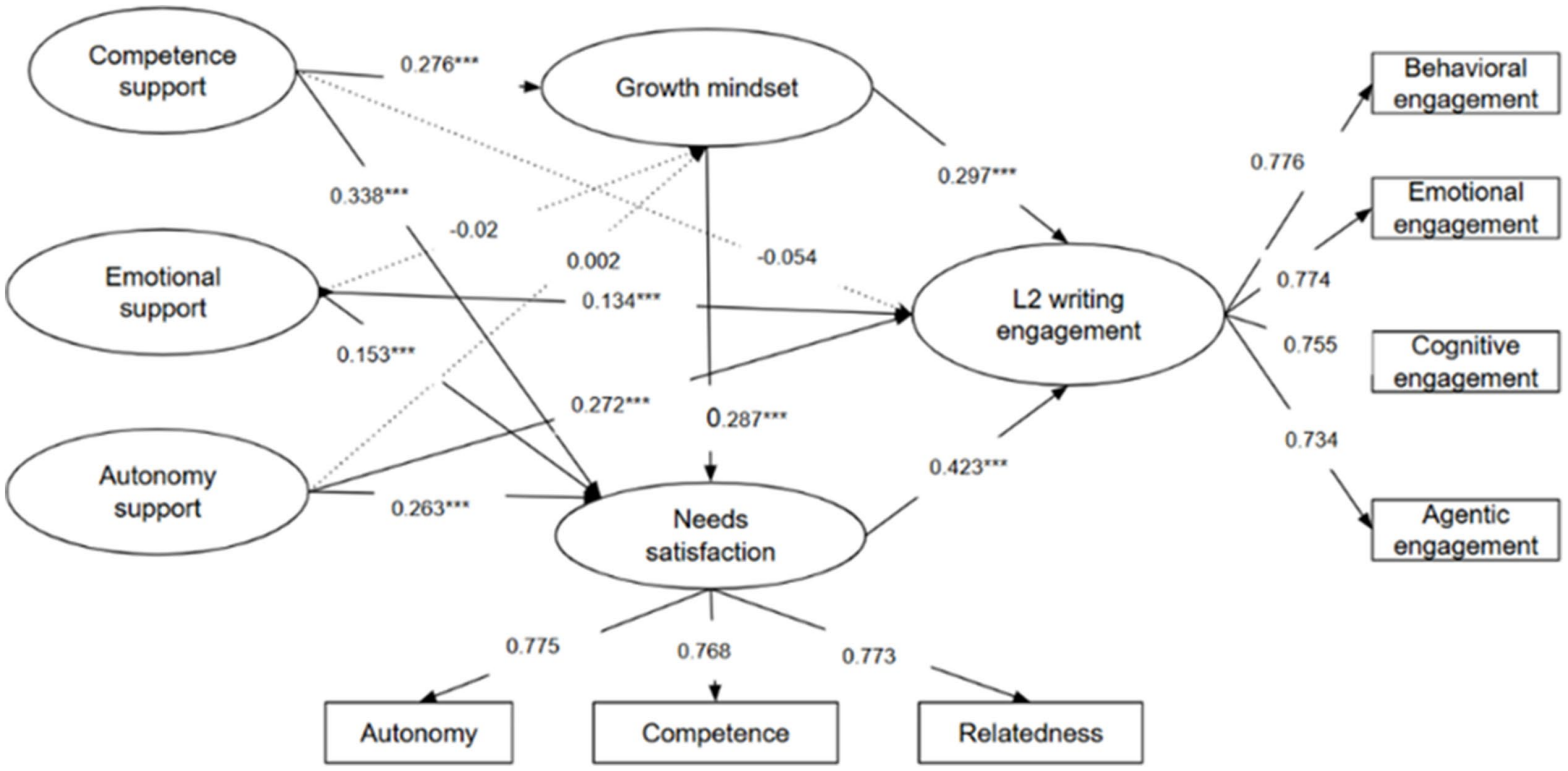
Final tested structural model. Standardized path coefficients are presented. ***Indicates *p* < 0.001.

The structural model also showed good fit, χ^2^/df = 1.642, CFI = 0.944, TLI = 0.939, RMSEA = 0.042, SRMR = 0.049. Figure 2 depicts the standardized path coefficients.

For the direct effects of teacher support on L2 writing engagement, emotional support (*β* = 0.134, *p* < 0.001) and autonomy support (*β* = 0.272, *p* < 0.001) showed significant positive association, whereas competence support did not show a significant association. Accordingly, H1b and H1c were supported, while H1a was not.

With respect to the effects of teacher support on growth mindset, competence support (*β* = 0.276, *p* < 0.001) significantly and positively predicted learners’ growth mindset, whereas emotional support and autonomy support were non-significant. For needs satisfaction, all three types of teacher support—competence (*β* = 0.338, *p* < 0.001), emotional (*β* = 0.153, *p* < 0.001), and autonomy support (*β* = 0.263, *p* < 0.001)—showed significant positive association. Growth mindset also positively predicted needs satisfaction (*β* = 0.289, *p* < 0.001). These findings indicate that both supportive teacher practices and students’ motivational beliefs contribute to fulfilling their basic psychological needs.

Finally, in predicting L2 writing engagement, both growth mindset (*β* = 0.297, *p* < 0.001) and needs satisfaction (*β* = 0.423, *p* < 0.001) showed significant positive effects. This suggests that learners who believe in their ability to improve and whose autonomy, competence, and relatedness needs are met tend to exhibit higher levels of engagement in L2 writing activities.

### 4.4 Mediation effect analysis

Following the path analysis, a bias-corrected bootstrapping procedure with 5,000 resamples and 95% confidence intervals was performed to examine the mediating roles of growth mindset and needs satisfaction. The mediation results are summarized in Table 3.

**Table 3:** Bootstrap analysis of indirect effects.

Mediation path	*β*	95% CI	*p*	Test results
H2a: CTS → GM → L2WE	0.082	[0.039, 0.123]	0.018	Supported
H3a: CTS → NS → L2WE	0.143	[0.087, 0.214]	0.006	Supported
H4a: CTS → GM → NS → L2WE	0.033	[0.020, 0.060]	0.002	Supported
H2b: ETS → GM → L2WE	−0.006	[−0.047, 0.026]	0.852	Not supported
H3b: ETS → NS → L2WE	0.065	[0.016, 0.118]	0.040	Supported
H4b: ETS → GM → NS → L2WE	−0.002	[−0.015, 0.012]	0.794	Not supported
H2c: ATS → GM → L2WE	0.001	[−0.033, 0.038]	0.896	Not supported
H3c: ATS → NS → L2WE	0.111	[0.059, 0.186]	0.010	Supported
H4c: ATS → GM → NS → L2WE	0.000	[−0.012, 0.013]	0.918	Not supported

Standardized coefficients (β) are based on 5,000 bootstrap samples. CI, confidence interval; CTS, competence teacher support; ETS, emotional teacher support; ATS, autonomy teacher support; GM, growth mindset; NS, needs satisfaction; L2WE, L2 writing engagement.

Regarding the mediating role of growth mindset, the indirect effect of the path “competence support → growth mindset → L2 writing engagement” was significant (*β* = 0.082, 95% CI [0.039, 0.123], *p* = 0.018), indicating that growth mindset significantly mediated the association of competence support and L2 writing engagement (supporting H2a). In contrast, the indirect effects of emotional support (*β* = −0.006, 95% CI [−0.047, 0.026], *p* = 0.852) and autonomy support (*β* = 0.001, 95% CI [−0.033, 0.038], *p* = 0.896) through growth mindset were not significant, suggesting that growth mindset serves as a meaningful mediator only for competence support (not supporting H2b and H2c).

For the mediating role of needs satisfaction, all three paths were significant. The indirect effect of competence support → needs satisfaction → L2 writing engagement was significant (*β* = 0.143, 95% CI [0.087, 0.214], *p* = 0.006) (supporting H3a). Emotional support also exerted a significant indirect effect via needs satisfaction (*β* = 0.065, 95% CI [0.016, 0.118], *p* = 0.040) (supporting H3b). Similarly, the path autonomy support → needs satisfaction → L2 writing engagement was significant (*β* = 0.111, 95% CI [0.059, 0.186], *p* = 0.010) (supporting H3c). These results indicate that needs satisfaction is a robust mediator through which competence support, emotional support, and autonomy support each enhance students’ L2 writing engagement.

The sequential mediation analysis further showed that the path “competence support → growth mindset → needs satisfaction → L2 writing engagement” was significant (*β* = 0.033, 95% CI [0.020, 0.060], *p* = 0.002) (supporting H4a). This pattern is consistent with the theorized SDT–CVT sequence, although the cross-sectional design precludes strong causal claims. In contrast, the sequential mediating effects were not significant for emotional support (*β* = −0.002, 95% CI [−0.015, 0.012], *p* = 0.794) (not supporting H4b) or autonomy support (*β* = 0.000, 95% CI [−0.012, 0.013], *p* = 0.918) (not supporting H4c).

### 4.5 Robustness analyses

To assess the robustness of the proposed motivational pathways, additional hierarchical regression analyses were conducted with GenAI literacy and GenAI attitudes entered as control variables. The inclusion of these variables did not explain additional variance in L2 writing engagement once growth mindset and needs satisfaction were accounted for (Δ*R^2^* = 0.001, Δ*F* = 0.015, *p* = 0.985). Importantly, the predictive effects of teacher support, growth mindset, and needs satisfaction remained stable.

## 5 Discussion

### 5.1 Competence-based teacher support as the primary predictor of growth mindset

The finding that only competence-oriented teacher support significantly predicted learners’ growth mindset suggests the importance of mastery-focused instructional cues in L2 writing. Similar patterns have been reported in traditional classroom settings, where competence support is related to adaptive beliefs about ability development by clarifying learning goals, standards, and strategies (Wang et al., 2021; Yin et al., 2025). In the present study, these processes are examined within GenAI-integrated L2 writing classrooms, where learners engage with writing tasks that require ongoing evaluation and revision.

Competence support directly communicates how writing ability can be improved through effort, strategy use, and feedback engagement. Instructional practices such as explaining quality criteria, modeling effective revision strategies, and providing process-oriented feedback help learners establish a clear link between effort and improvement, thereby strengthening growth-oriented beliefs about writing ability. In contrast, emotional and autonomy support, while motivationally valuable, do not explicitly convey mechanisms of skill development and therefore appear less directly aligned with the formation of a growth mindset in L2 writing.

The non-significant effects of emotional and autonomy support suggest that growth mindset functions as a task-specific epistemic belief, shaped more directly by instructional input that makes learning processes and standards of improvement explicit (Dweck, 2006; Yin et al., 2025). In the context of L2 writing, beliefs about the malleability of ability are thus more responsive to competence-oriented guidance than to affective reassurance or choice provision alone. In GenAI-integrated writing contexts, this tendency may be further amplified, as learners are required to critically evaluate, compare, and integrate feedback from both human and GenAI sources, rendering competence-relevant instructional cues particularly salient.

### 5.2 Perceived teacher support and growth mindset as predictors of needs satisfaction

All three dimensions of teacher support significantly predicted needs satisfaction, consistent with core propositions of Self-Determination Theory (Reeve, 2012; Ryan and Deci, 2024). This pattern indicates that when learners perceive their teachers as supportive of autonomy, competence, and relatedness, they are more likely to experience fulfillment of basic psychological needs during L2 writing. In the present study, these relationships are examined within the GenAI-integrated writing context.

Emotional support plays a central role in fostering relatedness satisfaction by promoting psychological safety and a sense of being understood during the writing process. Supportive teacher–student relationships may encourage learners to persist in challenging tasks and to engage more openly with feedback and revision. Autonomy support likewise contributes to needs satisfaction by affirming learners’ sense of volition and ownership over their writing decisions, enabling them to regulate their learning processes in accordance with personal goals and preferences. Competence support further enhances needs satisfaction by clarifying expectations, providing constructive feedback, and reinforcing learners’ perceptions of effectiveness and mastery in writing. Although teacher support and needs satisfaction are both assessed via learner self-report, they represent distinct constructs within SDT: the former reflects perceptions of instructional input, whereas the latter captures learners’ internal motivational experiences resulting from these conditions (Deci and Ryan, 2000; Reeve, 2012).

The growth mindset adds an important cognitive dimension to this process by shaping how learners interpret difficulty and feedback. Learners who endorse growth-oriented beliefs are more likely to view challenges as opportunities for improvement, which in turn supports sustained satisfaction of autonomy, competence, and relatedness needs. Together, these findings underscore the complementary roles of perceived teacher support and growth mindset in maintaining psychological need fulfillment during L2 writing, including in GenAI-integrated instructional contexts.

### 5.3 Perceived teacher support, growth mindset, and needs satisfaction as predictors of L2 writing engagement

Emotional and autonomy support showed direct effects on L2 writing engagement, highlighting the immediate motivational relevance of relational and volitional experiences in GenAI-integrated instructional contexts. Emotional support is associated with persistence by alleviating the vulnerability inherent in L2 writing and by providing psychological reassurance when learners navigate the uncertainties associated with GenAI-mediated feedback. Autonomy support showed a significant direct association with engagement by reinforcing learners’ sense of ownership over the writing process, including discretionary decisions regarding whether and how to incorporate GenAI tools, thereby sustaining behavioral involvement in technology-enriched settings. In contrast, competence support predicted engagement only indirectly through growth mindset and psychological needs satisfaction. Consistent with prior research (Guo et al., 2025; Yin et al., 2025), competence-oriented practices appear to influence engagement primarily after learners internalize enhanced self-beliefs rather than by eliciting immediate behavioral responses. In GenAI-mediated writing contexts, this indirect pathway may be particularly salient, given the heightened evaluative complexity associated with interpreting and integrating diverse feedback sources.

The mediation analyses underscore the centrality of needs satisfaction as the primary motivational mechanism linking teacher support to engagement. All three dimensions of support predicted engagement through needs satisfaction, reinforcing SDT’s proposition that autonomy, competence, and relatedness satisfaction are foundational drivers of persistence and cognitive investment (Ryan and Deci, 2024). Growth mindset mediated only the competence pathway, indicating that improvement beliefs are mainly associated with competence-related instructional cues. Competence support displayed the most complex motivational profile, activating both the mindset pathway and the full sequential mechanism (competence → growth mindset → needs satisfaction → engagement). The absence of sequential mediation for emotional and autonomy support suggests that these forms of teacher support do not primarily reorganize learners’ beliefs about how writing ability develops, which is a prerequisite for activating a mindset-driven sequence. Instead, because ETS and ATS mainly address learners’ immediate motivational conditions rather than belief formation, their influence on engagement is more likely to occur through direct, need-based processes without passing through growth mindset as an intermediate mechanism. This pattern highlights the unique role of competence support in strengthening learners’ evaluative capacities, facilitating need fulfillment, and fostering resilient engagement in an environment where feedback is diverse, abundant, yet imperfect.

In addition, robustness analyses showed that GenAI literacy and GenAI attitudes did not explain additional variance in L2 writing engagement once growth mindset and needs satisfaction were taken into account. This pattern suggests that, within the present model, learners’ engagement in GenAI-integrated writing appears to be more proximally associated with motivational appraisals and psychological need fulfillment (Ryan and Deci, 2020) than with general perceptions of GenAI-related competence or attitudes.

Taken together, these findings illustrate how established motivational processes operate in contemporary L2 writing classrooms where GenAI tools are present. Within such contexts, teacher support remains a central instructional and motivational resource, functioning through theoretically stable mechanisms that are consistent with prior research in non-GenAI classrooms.

## 6 Conclusion and contributions

This study investigated how teacher support, growth mindset, and needs satisfaction jointly shape L2 writing engagement in GenAI-integrated classrooms. Three key conclusions emerge. First, competence-oriented teaching was the only form of support that predicted improvement-focused beliefs, underscoring its central role in shaping students’ perceptions of the malleability of writing ability. Second, all three dimensions of teacher support—competence, emotional, and autonomy—together with learners’ beliefs, contributed meaningfully to psychological need satisfaction. Third, teacher support influenced engagement largely through two indirect routes, one via growth mindset and the other via needs satisfaction, with competence support additionally activating a sequential pathway in which growth mindset enhanced need satisfaction, which in turn promoted engagement.

By situating the SDT–CVT motivational sequence within GenAI-integrated classrooms, this study demonstrates the continued relevance of existing theory to a contemporary learning ecology in which human and GenAI feedback coexist. In addition, the findings advance understanding of teacher support by differentiating the functional roles of support dimensions: emotional and autonomy support exert direct effects on writing engagement, whereas competence support influences engagement indirectly through growth mindset and needs satisfaction. Finally, by integrating growth mindset into an SDT-based model, the study clarifies belief–motivation–engagement relations in L2 writing, showing that improvement beliefs are primarily associated with competence-oriented instructional cues.

Pedagogically, teachers should intentionally foster a growth mindset to sustain writing engagement by framing writing as a process of guided improvement. This can be supported through structured revision cycles, explicit instruction in feedback literacy, and classroom modeling of how to interpret, select, and refine GenAI-generated suggestions. In practice, emotional encouragement, autonomy-supportive task design, and competence-oriented scaffolding, such as clear performance criteria and exemplars, help teachers maintain pedagogical structure and sustain engagement in technology-rich L2 writing contexts when GenAI tools are available. Within this framework, teachers may strategically leverage GenAI to generate engaging prompts or instructional materials that enhance teaching quality and overall L2 writing outcomes.

## 7 Limitations

Despite the contributions of this study, several limitations warrant consideration.

First, the study relied on self-reported data, and GenAI integration was operationalized based on learners’ perceptions of instructional practices rather than on direct measures of usage frequency, specific tools, or the quality of teacher guidance. Future research could incorporate multi-source or usage-based evidence (e.g., GenAI-use analytics, classroom observations, or teacher reports) to more precisely capture how GenAI is implemented and how learners engage with human and GenAI feedback. In addition, because participants were drawn from multiple instructional contexts and classroom-level identifiers were unavailable, the findings should be interpreted at the individual level and should not be generalized to classroom- or teacher-level effects. Future research may further differentiate learners by year level, standardized English proficiency, and prior GenAI experience to examine potential heterogeneity in these motivational pathways.

Second, the cross-sectional design constrains causal inference. Although the proposed sequence aligns with SDT and CVT, longitudinal, experimental, or diary-based approaches are needed to verify temporal ordering and uncover potential reciprocal dynamics among teacher support, mindset, needs satisfaction, and engagement. Third, the study was conducted in a single cultural context, where norms surrounding teacher authority, instructional support, and technology acceptance may differ from those in more individualistic or technologically diverse settings. This limits the generalizability of the findings. Cross-cultural and cross-institutional research is needed to examine whether the motivational pathways observed here remain robust across varying pedagogical traditions and GenAI adoption patterns.

Finally, although teacher support was conceptualized as an environmental input and needs satisfaction as an internal motivational state, both constructs were assessed via learner self-report, and some degree of perceptual overlap cannot be entirely ruled out. Longitudinal or multi-source designs may further strengthen construct distinctiveness and provide a more rigorous test of the proposed motivational sequence.

## Data Availability

The raw data supporting the conclusions of this article will be made available by the authors, without undue reservation.
